# Neutron Time-of-Flight Spectroscopy

**DOI:** 10.6028/jres.098.006

**Published:** 1993

**Authors:** John R. D. Copley, Terrence J. Udovic

**Affiliations:** National Institute of Standards and Technology, Gaithersburg, MD 20899

**Keywords:** diffusion, hydrogen in metals, inelastic scattering, neutron choppers, neutron inelastic scattering, quasielastic neutron scattering, time-of-flight spectroscopy, tunneling spectroscopy, vibrational spectroscopy

## Abstract

The time-of-flight technique is employed in two of the instruments at the NIST Cold Neutron Research Facility (CNRF). A pulsed monochromatic beam strikes the sample, and the energies of scattered neutrons are determined from their times-of-flight to an array of detectors. The time-of-flight method may be used in a variety of types of experiments such as studies of vibrational and magnetic excitations, tunneling spectroscopy, and quasielastic scattering studies of diffusional behavior; several examples of experiments are discussed. We also present brief descriptions of the CNRF time-of-flight instruments, including their *modi operandi* and some of their more pertinent parameters and performance characteristics.

## 1. Introduction

The neutron time-of-flight technique has a long and distinguished history. In 1935, only 3 years after the discovery of the neutron, a pair of rotating disks was used to “prove by direct measurement that many of the slow neutrons [from a moderated Rn-Be source] are in the thermal velocity range” [1]. With the advent of the nuclear reactor (in 1942), more difficult measurements became possible, and a very different type of chopper was used by Enrico Fermi and his collaborators to determine the energy dependence of the absorption cross section of boron [2]. By 1960 relatively sophisticated phased chopper machines were being used to measure the scattering properties of materials such as water and polyethylene [3]. Much of the motivation for these experiments reflected the need for “scattering law” data for numerical calculations of the behavior of neutrons within nuclear reactors. Since the mid-sixties the emphasis has steadily shifted to the study of more and more complicated materials, and several kinds of time-of-flight instruments have been developed in order to make possible many different types of experiments [4,5].

The time-of-flight (TOF) method complements the triple-axis spectrometer (TAS) technique which is discussed elsewhere in this volume [6]. The TAS is ideally (but by no means only) suited to the study of excitations in oriented samples at specific points in (***Q***,***ω***) phase space. On the other hand TOF instruments may be used to explore rather large regions of phase space because many detectors simultaneously collect neutrons over a wide range of values of the scattered energy. The price paid for the much larger phase space volume is that the intensity on the sample is significantly reduced because the incident beam is pulsed. In another respect the TOF method complements the very high resolution backscattering and neutron spin echo techniques [7].

In Sec. 2 we present the basic principle of a time-of-flight measurement at a reactor-based spectrometer. We then illustrate several uses of the technique, describing a variety of applications in different fields of research. In Secs. 4 and 5 we describe the more important features of the TOF instruments at the CNRF, aspects of their anticipated performance, the concept of accessible regions in (***Q***,***ω***) space, and resolution and intensity considerations. In Appendix A we present important relationships between the wave- and particle-like properties of a thermal neutron. In Appendix B we discuss the functions of the various choppers in a multiple disk chopper TOF spectrometer.

## 2. The Time-of-Flight Technique

**Fig. 1:**
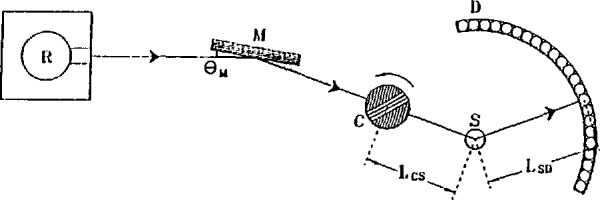
Schematic plan view of a simple time-of-flight spectrometer. The letters R, M, C, S, and D denote the reactor, monochromator, chopper, sample and detectors, respectively. Important distances are indicated. In practice, the slots in the (Fermi) chopper are curved in order to optimize its transmission.

A simple time-of-flight spectrometer is illustrated in Fig. 1. Neutrons from the reactor strike a crystal monochromator which is oriented at angle *θ*_M_ to the initial beam direction. Those with wavelength

$$\lambda_{0}=2d_{M}\;\sin\theta_{M},$$ (1)

where *d*_M_ is the spacing between reflecting planes in the monochromator, are Bragg reflected in the direction of the sample. The monochromatic beam, characterized by its energy *E*_0_ and wave vector ***k***_0_, is then pulsed by a chopper placed at a known distance *L*_CS_ from the sample. An array of detectors is arranged at a known fixed distance *L*_SD_ from the sample, and scattered neutrons arrive at the detectors at times determined by their scattered energies *E.* The time-of-flight of a neutron from the chopper to one of the detectors is simply

$$t_{\text{CD}}=t_{\text{CS}}+t_{\text{SD}}=\tau_{0}\;L_{\text{CS}}+\tau L_{\text{SD}}.$$ (2)

Here *t*_CS_ and *t*_SD_ are the times-of-flight of the neutron from chopper to sample and from sample to detector, respectively, and *τ*_0_ and *τ* are the reciprocal velocities of the neutron before and after scattering, respectively; relationships between λ, *τ*, *k*, and *E* are given in Appendix A. From Eq. (2) it is clear that *τ*, *E*, and the energy transfer

$$\hbar \omega =E_{0}-E,$$ (3)

may be determined from *t*_CD_ if λ_0_ is known. Given the angle between the incident and scattered neutron wave-vectors, i.e., the scattering angle *ϕ*, the wave-vector transfer

$$Q=k_{0}-k$$ (4)

is readily calculated. The vast majority of samples studied by TOF spectroscopy have no preferred orientation, so that all that generally matters is the magnitude of ***Q***. We shall ignore the vector character of ***Q*** in the remainder of this paper.

The basic quantity which is measured in a TOF experiment is a set of intensities *I*(*ϕ_i_*,*t_j_*) for *n*_D_ detectors located at scattering angles *ϕ_i_*(*i* = 1,2..*n*_D_), in *n_t_*, time channels centered at times *t_j_*(*j* = 1,2,..*n_t_*,) relative to an appropriate start time. Typically, measurements are made for the sample of interest and an “empty container,” and in some cases additional runs must be performed in order to establish calibration constants for the data analysis. The conversion of the raw data to a differential scattering cross section is straightforward, but corrections for instrumental resolution, and for effects [4] such as multiple scattering, sometimes complicate the data reduction procedure. The final result is the scattering function *S*(*Q*,*ω*) (cf. Ref. [8]) within the region of (*Q*,*ω*) phase space accessed in the experiment. The latter concept is discussed in Sec. 5.1.

## 3. Applications

The utility of TOF spectroscopy stems from its ability to access a region of (*Q*,*ω*) phase space which is at the same time important to an understanding of the chemical physics of condensed phase materials, yet difficult to explore by other spectroscopic techniques. Applications of TOF spectroscopy are diverse and numerous (see, e.g., Refs. [4] and [9]). They include investigations of (i) vibrational and magnetic excitations by inelastic scattering, (ii) intermolecular potentials by tunneling spectroscopy, and (iii) translational and reorientational dynamics by quasielastic neutron scattering (QENS). The theoretical framework behind such investigations is summarized elsewhere in this issue [7,8]. The following examples elaborate on these areas and provide a flavor of the benefits of TOF spectroscopy.

### 3.1 Inelastic Scattering

**Fig. 2:**
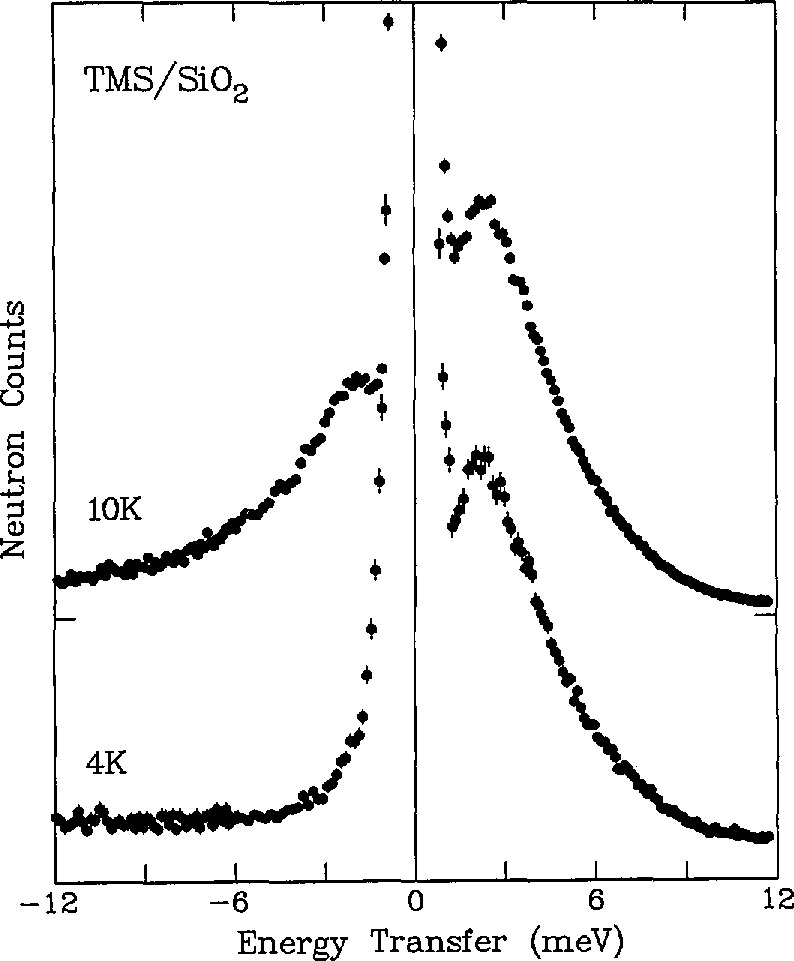
Low-energy (*E*_0_ = 13.8 meV) inelastie TOF speetra for trimethylsilyl adsorbates on silica at 4 and 10 K. Positive energy transfers correspond to neutron energy loss [11].

The TOF technique is useful as a low energy vibrational spectroscopic probe of condensed phase materials, particularly those in which hydrogen motions are important, due to the relatively high incoherent scattering cross section of hydrogen [4,8,10]. The energy range accessible in TOF measurements makes the technique complementary to other vibrational probes which access both higher and lower energy ranges. For example, Fig. 2 shows TOF spectra for trimethylsilyl [(CH_3_)_3_Si-, TMS] adsor-bates bonded to silica via surface oxygens [11]. An energy loss feature at ∼2.5 meV at 4 K shows up as both energy loss and energy gain features at 10 K due to increasing population of the 2.5 meV level above the ground state as the temperature is raised. This low energy feature is assigned to the torsional vibration of the entire trimethylsilyl group around the O-Si axis, since the relatively large moment of inertia of this group places the torsional mode in this energy range. The ∼ 2.5 meV vibrational feature is outside the higher energy (15–250 meV) window of a triple-axis/Be-filter spectrometer [10] which was used to measure the other TMS normal-mode vibrational energies.

**Fig. 3:**
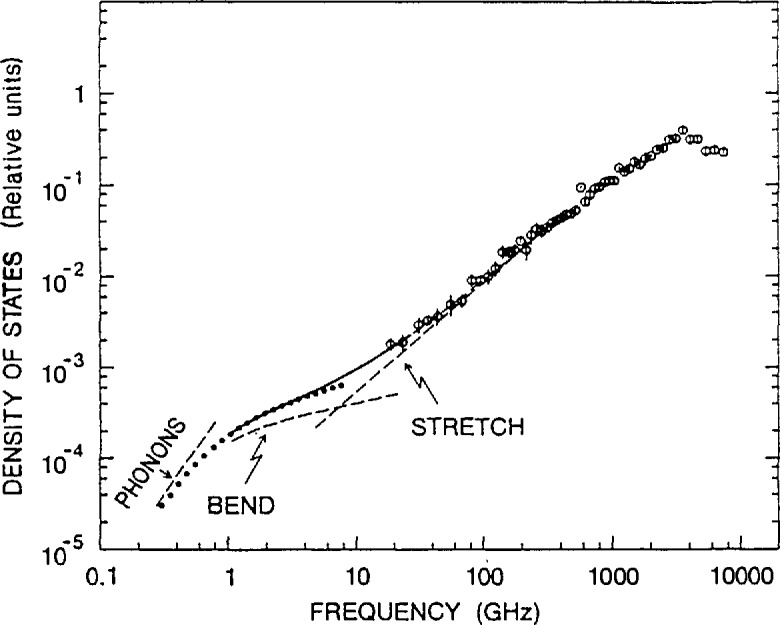
The density of states (DOS) of a silica aerogel synthesized by hydrolysis of tetramethoxysilane without the addition of ammonia to the reaetion water. The open circles are TOF measurements at 160 K using 8 Å incident neutrons. The dotted curve indieates the DOS that fits neutron spin-eeho data. The solid line is a fit to neutron backscattering data and is extrapolated as shown by the dashes throughout the high-frequency fracton region. The dashed lines indicate the asymptotic phonon as well as the independent bend and stretch eontributions [12].

The complementarity of TOF measurements with other vibrational probes is further illustrated in an investigation of the vibrational dynamics of fractal silica aerogels by neutron TOF, backscattering, and spin-echo techniques in conjunction with Raman spectroscopy [12]. Real fractal solids consist of particles, of typical size *a*, distributed in fractal fashion up to a correlation length *ξ*, beyond which the materials are homogeneous. Three régimes can be identified for vibrations of characteristic length *ℓ*, namely phonons (*ℓ > ξ*_ac_), fractons (ξ_ac_ > ℓ> *a*), and particle modes (*a >ℓ*), where *ξ*_ac_ is the acoustical correlation length. Figure 3 displays the density of states of a silica aerogel synthesized by hydrolysis of tetramethoxysilane without the addition of ammonia to the reaction water. The TOF data, which cover the high frequency region, were derived from the purely incoherent contribution using the difference between signals measured on two samples: one with protons attached to the silica network and the other with the protons substituted by deuterons. The combination of different neutron scattering measurements illustrates the three régimes (note that 1 GHz≃4.1357 μeV): phonons at low frequencies, (< 0.7 GHz), fractons at medium frequencies (0.7−250 GHz), and particle modes at higher frequencies (>250 GHz). In addition, the data suggest two distinct elastic régimes in the fracton range as previously predicted [13], associated with bending at smaller scales and stretching at larger scales. Greatly different Debye-Waller factors were found in the two régimes, enabling them to be unambiguously distinguished in the neutron spectra.

**Fig. 4:**
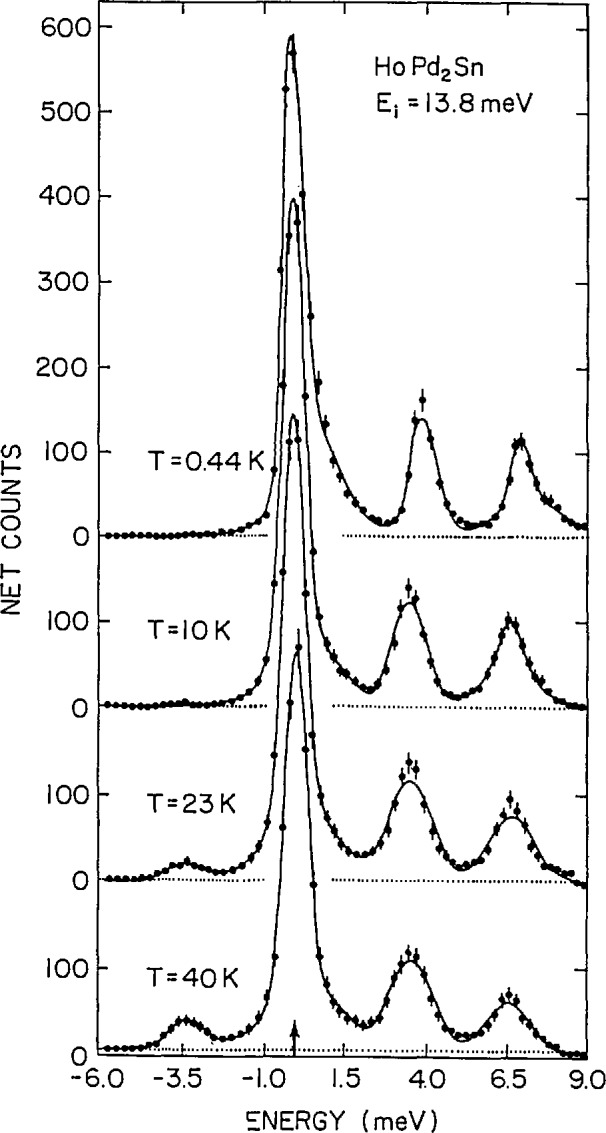
Elastic and inelastic scattering observed in a powder sample of HoPd_2_Sn using the TOF technique with incident energy 13.8 meV. To improve the statistics, the spectra shown were obtained by summing over data acquired in 60 detectors spanning a *Q* range from 0.48 to 3.85 Å^−1^. At 23 and 40 K, transitions from higher-energy to lower-energy levels are also evident in neutron-energy-gain (*E* < 0) [14].

Time-of-flight instruments, unlike crystal spectrometers [6], do not readily lend themselves to investigations at constant wave-vector transfer *Q.* On the other hand the broad *Q* range available at a given energy transfer, using a multidetector TOF spectrometer, is well-suited to the study of dispersionless phenomena such as crystal-field excitations [14]. This is exemplified in Fig. 4, which illustrates the crystal field transitions (at 3.52 and 6.65 meV at 10 K) of the rare earth ion Ho^3+^ in the cubic Heusler alloy HoPd_2_Sn. The neutron energy loss intensity for each transition decreases with increasing temperature as expected due to the depopulation of the crystal-field ground state. At elevated temperatures, when the higher energy crystal-field levels become thermally populated (e.g., at *T* = 23 and 40 K), an additional transition at −3.5 meV on the neutron energy gain side is also evident. At each temperature, no variation of the excitation energies was observed from detector to detector indicating that the transitions were indeed dispersionless. This permitted an improvement of the statistics by summing many detectors over a broad *Q* range.

**Fig. 5:**
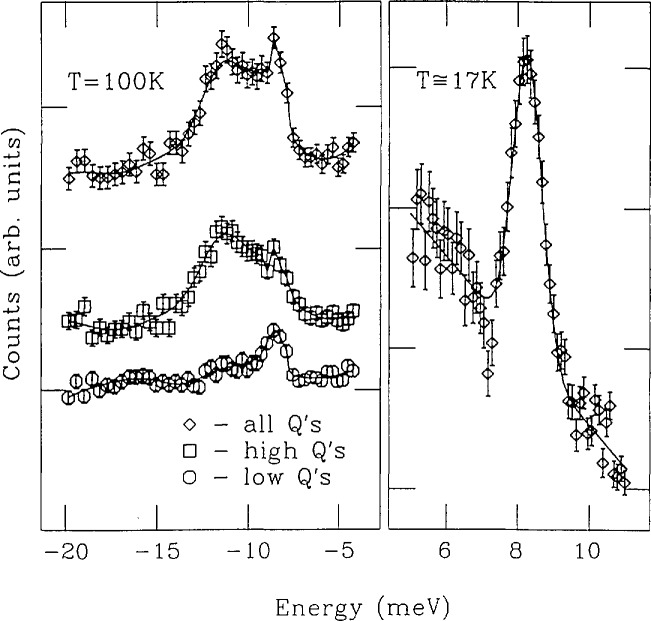
Examples of inelastic TOF spectra (*E*_0_=13.8 meV) for Zn_0.94_Co_0.06_S. The right panel shows the neutron energy-loss side of the spectrum obtained at 17 K with a fitted Gaussian line shape. The left panel shows energy-gain spectra at 100 K obtained by summing the counts from all detectors, from the “high-*Q*” detectors, and from the “low-*Q*” detectors. The curves are guides to the eye [15].

Figure 5 illustrates the dispersionless, first excited level, magnetic-pair scattering energy (*E*_NN_ = 8.19 ± 0.05 meV) in both energy loss (at 17 K) and energy gain (at 100 K) for nearest-neighbor (NN) Co^2+^ ions in the II–VI diluted magnetic semiconductor Zn_0.94_Co_0.06_S [15]. The interaction between the two NN Co^2+^ ions can be treated, to a first approximation, as a Heisenberg-type exchange for a pair of *S_i_ = S_j_* =3/2 spins described by the Hamiltonian *H = 2J*_NN_*S_i_*·*S_j_.* Thus *E*_NN_ becomes a direct measure of the antiferromagnetic exchange constant *J*_NN_ (where *E*_NN_= 2*J*_NN_), yielding a value of *J*_NN_ = 4.10 ± 0.03 meV, in good agreement with the result obtained from magnetic susceptibility studies [16]. In this alloy, the magnetic Co^2+^ ions are randomly distributed over a face-centered-cubic cation sublattice. Hence, 48% of the ions are singlets, 24% are members of NN pairs, 4% belong to triads, and the remainder to larger clusters. Since the singlet Co^2+^ ions only possess excited state levels with much higher transition energies outside the relevant measurement range, Co-Co pair scattering is the dominant magnetic inelastic effect in this alloy composition region. Yet its intensity is expected to be relatively weak because the pairs constitute only a small fraction of the total number of atoms in the sample. Since pair scattering energies are dispersionless, the ability to analyze the 100 K TOF spectra as a function of the *Q* range is useful in order to corroborate evidence that the broad feature at −11 meV is due to *Q*^2^−dependent phonon scattering, rather than to magnetic-pair scattering.

### 3.2 Tunneling Spectroscopy

**Fig. 6:**
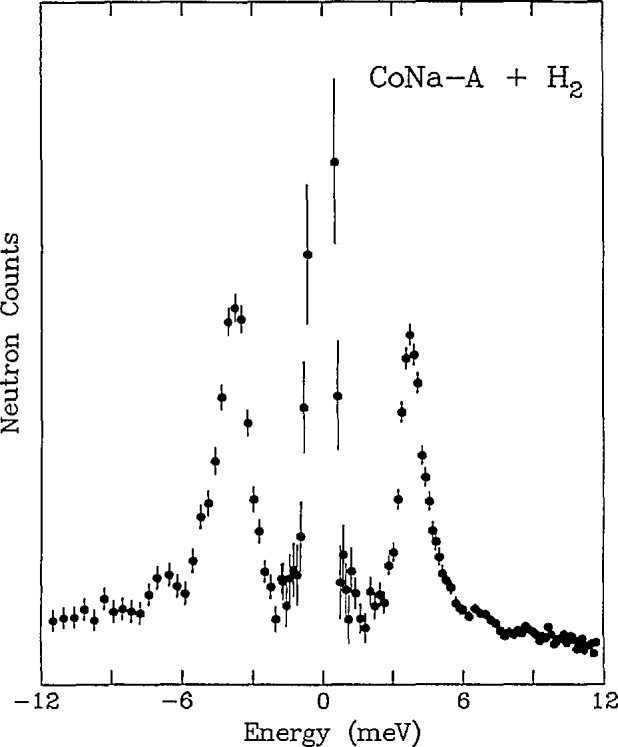
TOF spectrum (*E*_0_ = 14.8 meV) of 0.5 molecules of hydrogen per supercage adsorbed on Co._4.1_Na_3.8__−_A zeolite at 12 K. The spectrum of the dehydrated zeolite has been subtracted from the data. Positive energy represents neutron energy loss [17].

Time-of-flight spectroscopy is a useful probe of intermolecular potentials through characterization of tunneling transitions in condensed-phase materials. For example, Fig. 6 is a TOF spectrum illustrating the rotational tunnel splitting of the librational ground state of molecular hydrogen adsorbed in the cavities of partially cobalt-exchanged type A zeolite (C_04.1_Na_3.8__−_A) [17]. The assignment of the 3.8 meV neutron energy gain and energy loss features to the librational ground-state splitting (i.e., between the J = 0 (para H_2_) and *J* = 1 (ortho H_2_) rotational states) is unambiguous since the expected intensity ratio between energy gain and energy loss processes would be about 1:40 at 12 K for a translational excitation. This assignment, in conjunction with excitation data at higher energy transfer determined using a triple-axis spectrometer, is in good agreement with a model for the H_2_ molecules in a twofold cosine potential with two degrees of rotational freedom. The model implies that the H_2_ molecules are bound end-on to the cobalt cations, and perform 180° reorientations with a barrier of 55–68 meV.

**Fig. 7:**
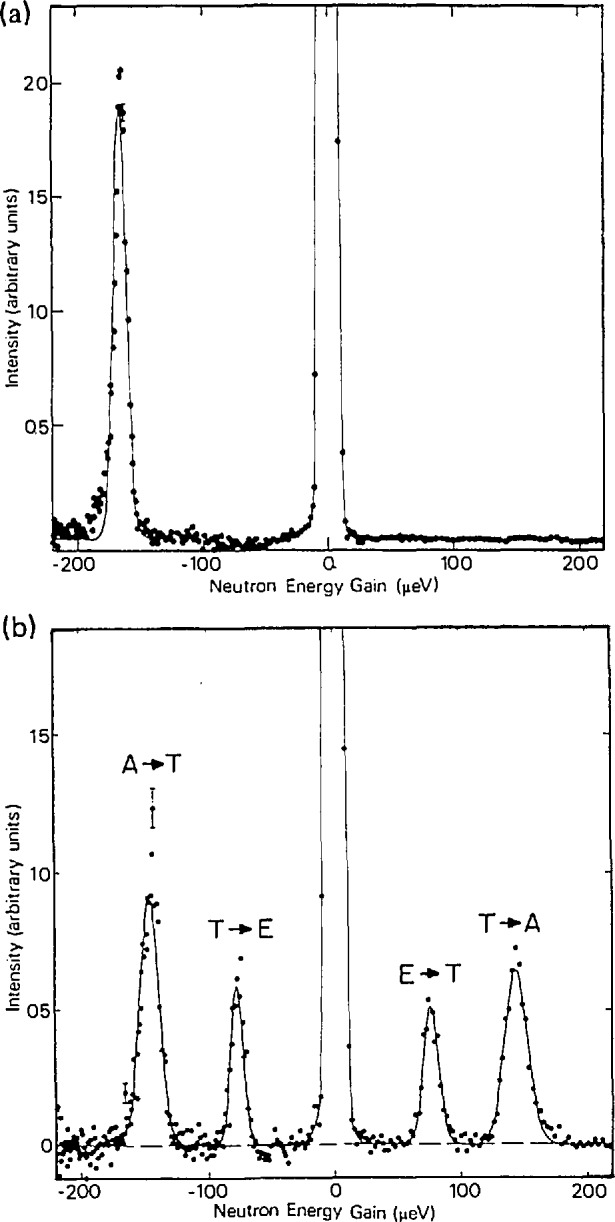
TOF spectra for 13 Å neutrons scattered by CH_4_ (phase II) at sample and spin temperatures of (a) < 0.2 K and (b) 5 K. The A−T and E−T transitions are labelled in (b) [18].

**Fig. 8:**
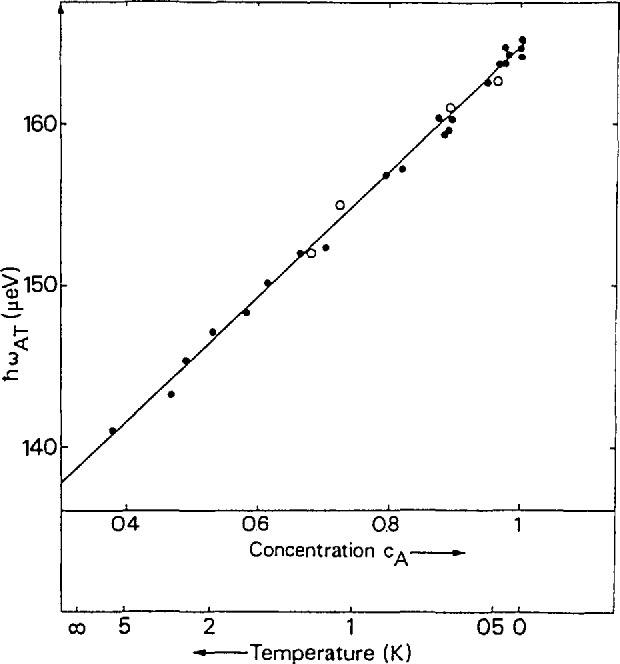
Energy of the A–T transition peak as a function of the concentration of the A spin species. The straight line represents a fit with the model of inhomogeneous broadening due to a changing mixture of spin states [18].

The advent of high resolution TOF (and backscattering [7]) spectrometers, over the past two decades, has provided the ability to measure much lower energy tunneling transitions of condensed molecules and atoms. This is exemplified by the study of quantum states, transitions and interactions in the solid methanes [18]. Figure 7 illustrates TOF tunneling spectra for phase II CH_4_ at low temperatures. In this cubic phase (space group *Fm3c*), two of the eight molecules in the unit cell are essentially freely rotating, while the other six molecules are in sites of $\left(\bar{4}2m\right)$ symmetry, are orientationally ordered, and undergo tunneling. There are three corresponding tunneling states for a tetrahedral molecule or group experiencing a hindering potential in a tetrahedral site symmetry: a singlet ground state (A) and triply and doubly degenerate levels (T and E). Neutron induced transitions from A to E states are not observed since this requires a nuclear spin change from *I = I* to *I* = 0, which cannot be caused by a spin 1/2 particle. The 0.2 K spectrum shows an almost complete occupation of the A ground state species, which allows observation of A−T transitions only. As the temperature is raised both T and E states become populated, allowing observation of A–T and E–T transitions by both neutron energy loss and energy gain scattering. An excellent fit to the observed temperature-dependent widths and energies of these transitions (Fig. 8) assumes a continuously changing local potential, reflecting the changing mixture of spin states with temperature. The highest A–T energy (163.5 µeV) observed at the lowest temperatures (< 0.5 K) reflects the potential for A level species surrounded by like species. Decreasing frequencies (and concomitant increasing linewidths), observed with increasing temperature, indicate an increasing admixture of T and E species, increasing the average barrier height of the ordered CH_4_ molecules in the unit cell.

### 3.3 Quasielastic Neutron Scattering (QENS)

**Fig. 9:**
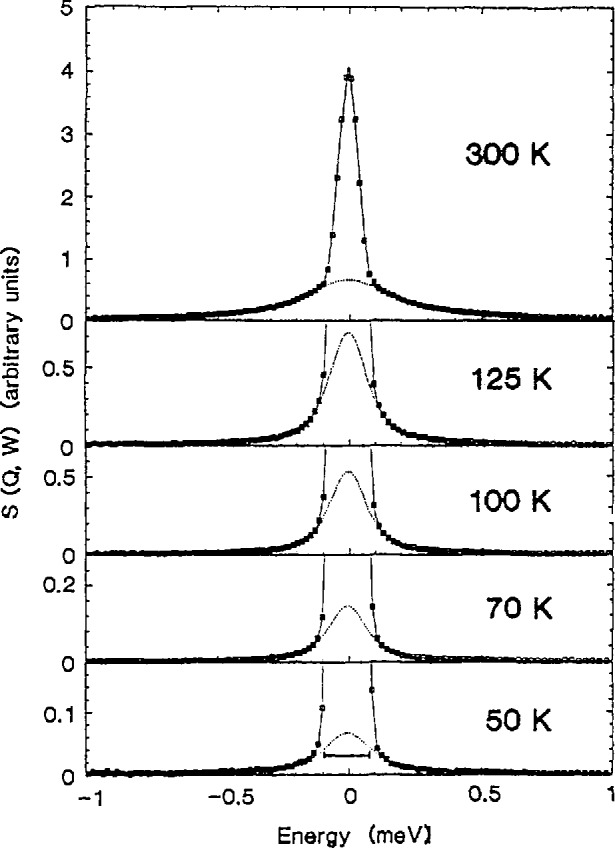
Quasielastic neutron scattering spectra for *α*-ScH_0.16_ at several temperatures at 70 µeV FWHM elastic energy resolution. The solid lines are the results of least-squares fits to the data; the dotted lines represent the Lorentzian quasielastic component. The increase in the quasielastic linewidth at low temperature is illustrated in the 50 K spectrum, where the length of the horizontal bar is equal to the width of the 70 K Lorentzian component [19].

**Fig. 10:**
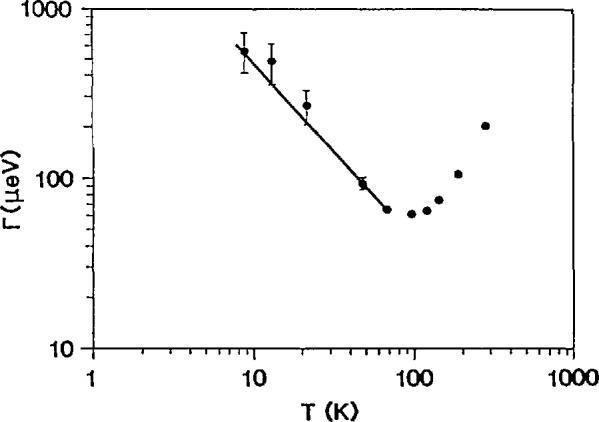
Fitted Lorentzian linewidths (FWHM) for *α*-ScH_0.16_. The solid line is the fit to the data below 100 K, assuming electronically induced linewidth broadening with decreasing temperature as described in the text [19].

Time-of-flight spectroscopy is well-suited to probing the translational and reorientational dynamics of atoms and molecules in condensed phase materials, particularly those with important hydrogen motions, via the *Q*- and T-dependence of the associated quasielastic neutron scattering. For instance, the localized motion of hydrogen in an *α*-ScH_0.16_ solid solution has been investigated by QENS using TOF techniques [19]. The hydrogen atoms in this system are restricted to pairs of nearest-neighbor tetrahedral (t) sites between metal atoms along the *c* -axis. All scattering spectra were fit with an empirical two-component function comprising an elastic term described by the resolution function of the spectrometer and a resolution-broadened Lorentzian quasielastic term. Throughout the measurements, the invariance of the quasielastic linewidth *Γ* with *Q* corroborated the localized nature of the hopping motions. A hopping distance of 0.10 nm (1 Å) was abstracted from the data on the elastic incoherent structure factor (EISF) as a function of *Q*, consistent with the known nearest-neighbor t−t distance in this material. Figure 9 illustrates quasielastic scattering spectra for *α*-ScH_0.16_ at several temperatures; Fig. 10 is a plot of *Γ* vs *T.* The data show hydrogen hopping rates between t sites exceeding ∼7 × 10^10^ s^−1^ at all temperatures, indicating very rapid motion compared with the bulk diffusion rate in these systems. The apparent hopping rate increases to 10^12^ s^−1^ at 10 K after passing through a minimum at ∼ 100 K. This remarkable upturn of *Γ* below the minimum is approximately proportional to *T^−^*^1^ in the range shown and is explained in terms of Kondo’s [20] prediction of nonadiabatic effects of the coupling of the metal conduction electrons to the protons.

**Fig. 11:**
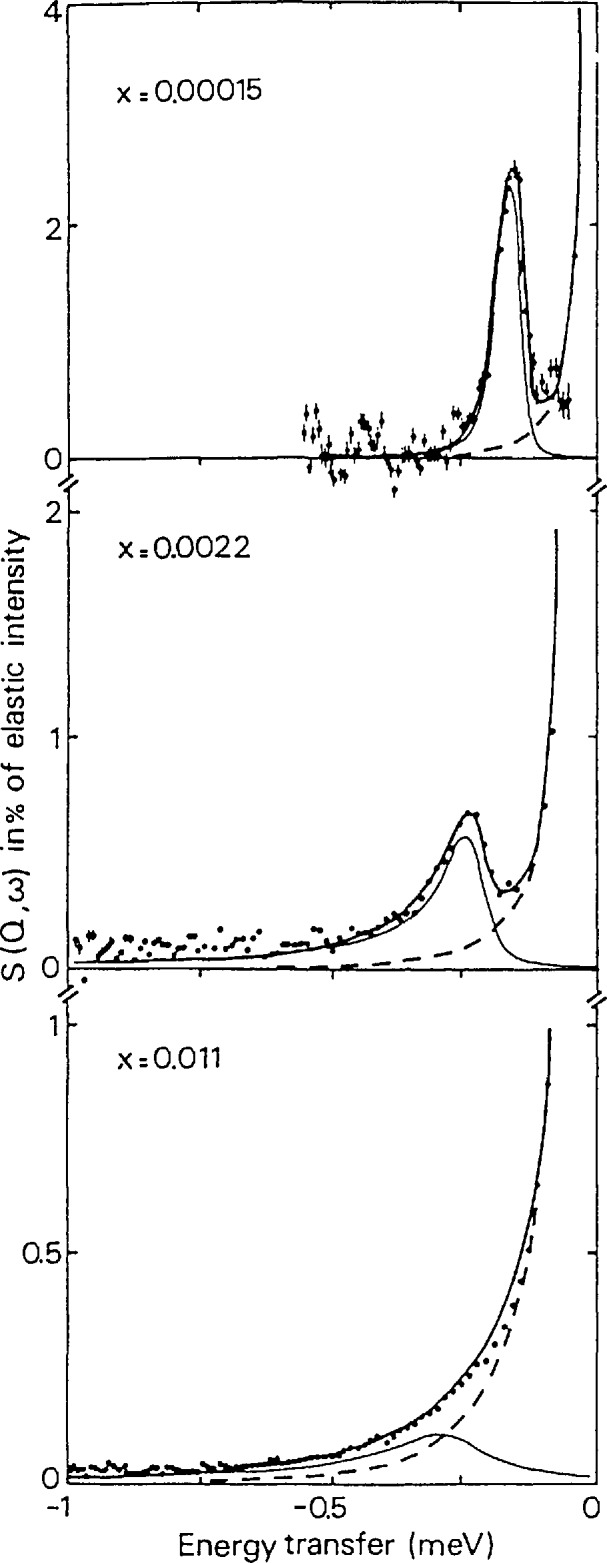
TOF tunneling spectra measured (in neutron energy loss) at three OH concentrations in Nb(OH)*_x_* at 1.5 K. Elastic energy resolution was 55 μeV FWHM [21].

A similar Kondo effect was observed below ∼70 K in the quasielastic scattering from hydrogen trapped by oxygen impurities in Nb (i.e., Nb(OH)*_x_*, 1.5 × 10^−4^⩽ *x* ⩽ 1.1 × 10^−2^) [21,22]. In particular, the hydrogens are trapped at weakly distorted tetrahedral sites in the body-centered-cubic lattice generated by the presence of oxygen defects at octahedral interstitial sites. Local diffusion of hydrogen occurs between at least two nearest-neighbor tetrahedral trap sites. Above ∼70 K, the diffusion is dominated by the interaction with phonons. Below 5 K, well-defined tunneling eigenstates exist due to derealization of the H between trap sites. Figure 11 shows a narrow inelastic tunneling transition at the lowest measured hydrogen concentration (*x* = 1.5 × 10^−4^), which changes into a broad peak at higher concentrations due to increasingly strong interactions between defects.

**Fig. 12:**
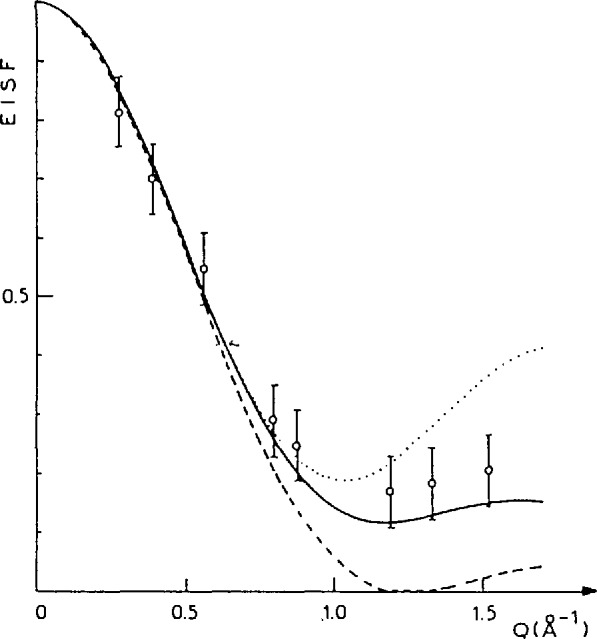
Elastic incoherent structure factors (EISFs) of benzene adsorbed in Na-mordenite zeolite as a function of scattering vector *Q.* Open circles represent the experimental data. Theoretical EISFs are shown for rotational diffusion (broken line), for uniaxial rotations of 2*π*/6 (full line) and for uniaxial rotations of 2*π*/3 (dotted line) [23].

The ability of TOF techniques to probe molecular dynamics on an atomic scale is further exemplified by QENS investigations of the translational and rotational motions of hydrocarbons adsorbed in zeolites, namely benzene in Na-mordenite [23] and methane in Na-ZSM-5 [24]. Figure 12 compares the EISF data for benzene in Na-mordenite with various theoretical models for the benzene reorientation. The data suggest that the benzene molecules, likely adsorbed by the Na cations via cation interaction with the *π*-electrons, undergo discrete uniaxial rotational jumps of 2*π*/6. In contrast, methane in Na-ZSM-5 is found to undergo isotropic rotational diffusion. In this model, methane molecules are assumed to perform continuous small-angle rotations and therefore have no preferred orientation in space. This rotational motion is found to be much slower for methane in the zeolite than in physisorbed layers or in the solid phase.

**Fig. 13:**
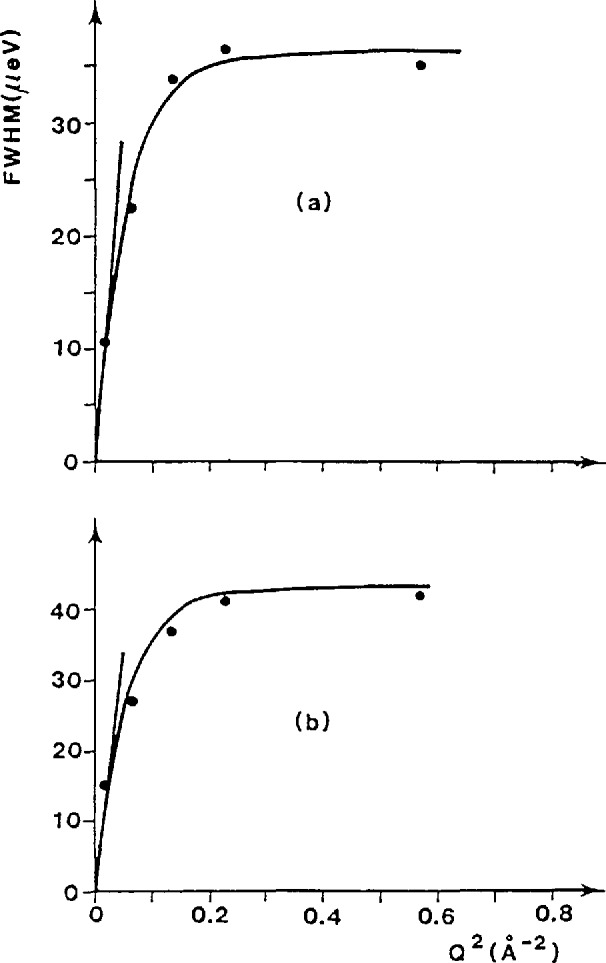
Broadening of the elastic peak as a function of *Q*^2^ for methane in ZSM-5 zeolite at 250 K: (a) 1.5 molecules and (b) 2.8 molecules per unit cell [24].

The translational diffusion behavior also differs between the two hydrocarbon/zeolite systems. For benzene in Na-mordenite, the benzene-Na bonding is weak, and the elastic peak width possesses a *Q*^2^-dependence, implying that the benzene molecules follow Fick’s law of continuous translational diffusion, characteristic of diffusion in a liquid, rather than a jump diffusion mechanism. The translational diffusion coefficient was found to be 6.7 × 10^−7^ cm^2^ s^−1^ at 300 K. In contrast, for methane in Na-ZSM-5, the broadening of the elastic peak versus *Q*^2^ deviates from a straight line (see Fig. 13) so that, on an atomic scale, the motion of methane is not simply Fickian. Instead the data agree with a model for translational diffusion which assumes jump diffusion with a Gaussian distribution of jump lengths. The physical interpretation of the model is that methane can perform small jumps inside the zeolite channels, but larger jump distances across the channel intersections are also possible. After a large number of jumps, Fickian diffusion can be observed. The diffusion coefficients for long range translational motion (2.7 × 10^−5^ and 5.5 × 10^−5^ cm^2^ s^−1^, at 200 and 250 K, respectively), determined from asymptotic values of the broadening at low *Q*, do not vary much with methane loading.

## 4. Time-of-Flight Instruments at the Cold Neutron Research Facility

Two time-of-flight spectrometers are planned for the guide hall of the CNRF. The first of these instruments, which is primarily designed for medium resolution applications, is a modified version of the type of instrument depicted in Fig. 1; it is located on guide NG-6, as shown in Fig. 7 of Ref. [25]. We call it the Fermi Chopper Spectrometer (FCS).

The second instrument, to be located at guide NG-4, uses a number of disk choppers to monochromate and pulse the incident beam; it is intended for high resolution measurements but may also be operated with relaxed resolution when required. We call it the Disk Chopper Spectrometer (DCS).

### 4.1. The Fermi Chopper Spectrometer

**Fig. 14:**
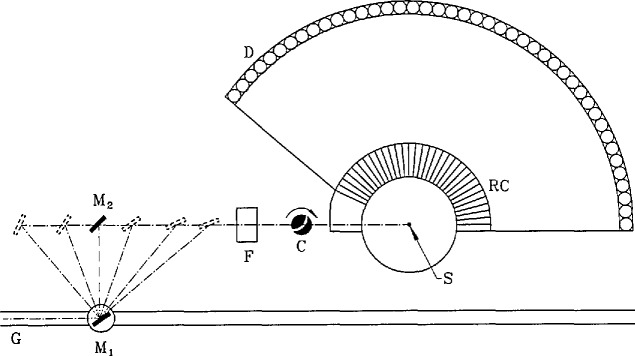
Simplified plan view of the Fermi chopper spectrometer. The letters G, F, C, S, and D dcnote the guide, filter, chopper, sample and detectors respectively; M_1_ and M_2_ are the monochromator crystals and RC is the (oscillating) radial collimator. Incident wavelengths between 0.23 and 0.61 nm are obtained by modifying the Bragg diffraction angle of the double monochromator, as suggested by the dashed lines in the figure. The guide is continued to additional instruments downstream.

The Fermi Chopper Spectrometer is illustrated schematically in Fig. 14. Detailed specifications are listed in Table 1. The incident beam wavelength (0.23−0.61 nm) is determined using a double monochromator. The principle of this device is similar to that of a single monochromator, but an important advantage is that the selected neutron wavelength can be changed without having to rotate virtually the entire spectrometer about the monochromator axis. This significantly simplifies the design of the instrument. The monochromators are made of individually aligned pyrolytic graphite (PG(002)) crystals. A 60′ Soller collimator is located between the monochromators. The first monochromator is flat, whereas the second monochromator is vertically curved in order to focus intensity at the sample position. Vertical focussing is optimized when the vertical mosaic spread is minimized, yet this same mosaic spread in the horizontal direction would lead to an unnecessarily small wavelength spread and a consequent decreased intensity on the sample. Hence each monochromator is made of two layers of crystals, each layer possessing a 25′ mosaic, but staggered horizontally with 25′ angular offset. This effectively yields a more desirable anisotropic mosaic distribution, 25′ vertically and 50′ horizontally.

The neutron beam leaving the second monochromator is filtered, using pyrolytic graphite or liquid nitrogen-cooled beryllium (see Table 1), in order to remove Bragg (*λ*/*n*) (where *n* is an integer >1) order contamination (cf. [6]), as well as epithermal neutrons. It is then pulsed using a “Fermi chopper” [2]. This is a device which spins about a vertical axis (normal to the direction of the beam), and is fitted with a set of curved slots. The curvature of the slots and the speed of the chopper determine the optimum transmitted wavelength of the chopper [5]. Two slot packages are available, corresponding to optimum transmitted wavelengths of 0.4 and 0.15 nm, respectively, at close to the maximum chopper speed.

The sample chamber can accommodate a wide variety of cryostats and furnaces. An oscillating radial collimator between the sample and the detectors blocks most of the scattering from material components which surround the sample (e.g., heat shields of cryostats). The evacuated sample-to-detector flight path contains an array of ^3^He neutron detectors covering a scattering range of 5°–140°. Signals from the detectors are amplified, shaped and filtered, and then fed to a time-of-flight encoder and CAMAC-based histogramming memory. The contents of this module are periodically transferred to a microVax computer for subsequent analysis.

### 4.2 Disk Chopper Spectrometer

**Fig. 15:**
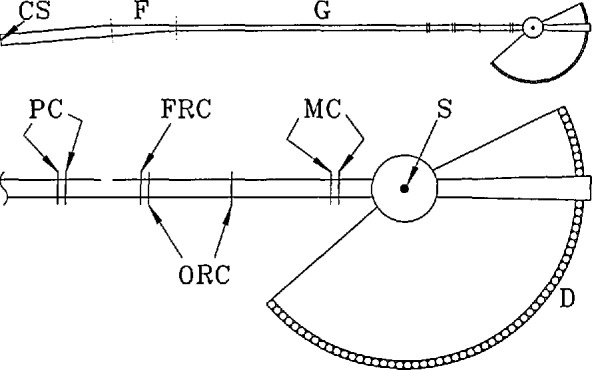
Simplified plan view of the disk chopper spectrometer. The upper diagram shows the overall setup of the instrument including cold source (CS), filter (F), and guide (G). The lower diagram shows the choppers, the sample position (S), and the detectors (D); PC, ORC, FRC, and MC denote pulsing, order removal, frame removal, and monochromating choppers, respectively. The width of the guide, the separation between closely spaced choppers, and the angle between initial and final guide sections, have been exaggerated for clarity. The filter is a tapered section of guide, ∼7 m long, that redirects slow neutrons (through 0.25°) whereas fast neutrons and *γ* rays are removed from the beam because they are not reflected. It is located within the wall between the reactor confinement building and the guide hall.

**Fig. 16:**
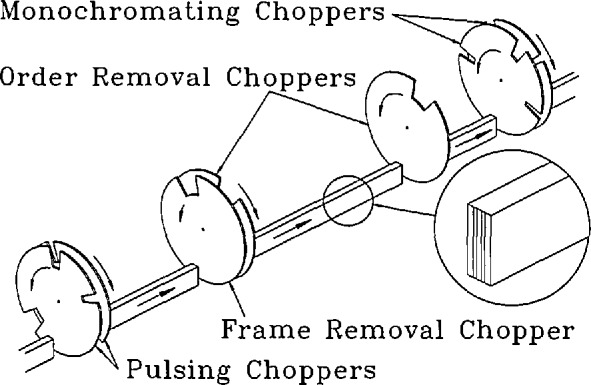
Simplified view of the choppers and guide for the disk chopper spectrometer. Note that the pulsing and monochromating chopper disks each have three slots (of different sizes for different resolution modes of the instrument) and that the guide is divided into five channels.

The Disk Chopper Spectrometer is illustrated schematically in Fig. 15. The “front end” comprises a novel neutron filter followed by a total of seven disk choppers which collectively produce a clean, usable, pulsed, monochromatic neutron beam at the sample position. The purpose of each of these choppers is explained in Appendix B. The multichopper design (Fig. 16) allows the user a wide and continuous choice of wavelengths, basically constrained by the available spectrum of neutrons in the guide; a change in wavelength is achieved by changing the phase relationship between the disks.

In general the instrumental resolution may be varied (at fixed wavelength) either by changing the speed of the choppers or by changing the width of the beam. It is actually preferable, for reasons discussed in Appendix B, to change the width of the beam keeping the choppers spinning as fast as possible consistent with the desired resolution and with safe operating procedures. To optimize performance this implies changes both in the widths of the slots in the disks and in the width of the guide [26]. The practical realization of this option requires some explanation.

Each of the disks in the pulsing and monochromating chopper pairs is equipped with three slots of different width, as shown in Fig. 16. The slots in each counter-rotating pair are located such that the width of the slot presented to the neutron beam can be changed by grossly changing the relative phasing of the disks. The choice of slot positions is complicated because of effects resulting from the small separation between the members of a counter-rotating pair [27,28].

The neutron guide for the DCS is of rectangular cross section, 150 mm high and 60 mm wide, until it reaches the filter; thereafter it is 30 mm wide. After the first chopper it is best described as a channeled guide, fitted with internal reflecting plates as shown in the insets to Figs. 15 and 16. Beam-defining masks limit the number of channels which transmit neutrons, effectively changing the width of the guide. The widths of the channels, and the widths of the slots in the disks, have been chosen to optimize intensity on the sample under each of three distinct resolution conditions at a given wavelength and chopper speed [26]. Typically the overall resolution width doubles, and the intensity on the sample increases by about an order of magnitude, in going from high to medium resolution or from medium to low resolution. The capability to change resolution at fixed wavelength and fixed chopper speed is only possible because we use counter-rotating choppers with multiple slots [29].

After the final chopper the layout of the instrument is not unlike that of the Fermi Chopper Spectrometer, described in the previous subsection. The sample chamber is comparable in size and will be evacuated. On the other hand, the flight path between sample and detector will be filled with an inert gas in order to reduce scattering, and the detectors will be mounted externally. An important difference is that the distance *L*_SD_ is significantly larger (4000 mm), in order to achieve the desired energy resolution. For the same reason, the detectors will be very thin (∼10 mm) in the direction travelled by the neutrons, but relatively wide (∼32 mm) in order to capture as many neutrons as possible. They will be rectangular ^3^He tubes, typically 400 mm long, and there will be three detector banks spanning a wide range of scattering angles. The data acquisition system will be an expanded and somewhat more elaborate version of the system used in the FCS.

Certain specifications of the DCS are listed in Table 2; some of the numbers may be modified as the design progresses. Figures for the anticipated intensity on the sample are somewhat speculative, and the reader is cautioned not to take these intensities at face value. The *Q* resolution of the instrument will depend on how the detectors are grouped; the best achievable resolution can be estimated from the anticipated resolution in *λ*_0_ (which depends on *λ*_0_ itself, the speed of the choppers, and the resolution mode of the choppers), the divergence of the incident beam (which depends on *λ*_0_), and the angle subtended by a single detector at the sample position.

## 5. Experimental Considerations

When planning a time-of-flight experiment the interplay of resolution and intensity requirements is an important concern. Generally speaking, instrumental resolution improves dramatically with increasing wavelength; for example the energy width of the elastic scattering approximately varies as the *n* th power of *E*_0_, where *n* is about 3. On the other hand the intensity at the sample position tends to decrease as the incident wavelength is increased; for instruments on guide tubes the intensity varies as λ^−3^ at long wavelengths. It is therefore necessary to strike a compromise between the conflicting requirements of high intensity and good resolution.

A further disadvantage of long wavelengths is that an increase in wavelength also reduces the accessible region in (*Q*,*ω*) space. Generally speaking the best policy is to use the shortest wavelength consistent with the desired instrumental resolution, in order to be able to access as large a region of (*Q*,*ω*) space as possible.

### 5.1 Accessible Regions in (*Q*,*ω*) Space

In TOF experiments the accessible region depends on the choice of incident energy and on the placement of the detectors. The cosine rule, applied to the vectors ***Q, k***_0_ and ***k*** [(cf. Eq. (4)], yields the result

$$Q^{2}=k_{0}^{2}+k^{2}-2k_{0}k\cos\left(\phi \right).$$ (5)

Converting wave-vectors to energies (see Appendix A), and using Eq. (3), Eq. (5) may be rewritten as follows:

$$\hbar^{2}Q^{2}/2m=2E_{0}-\hbar \omega -2\sqrt{E_{0}\left(E_{0}-\hbar \omega \right)}\cos\left(\phi \right).$$ (6)

**Fig. 17:**
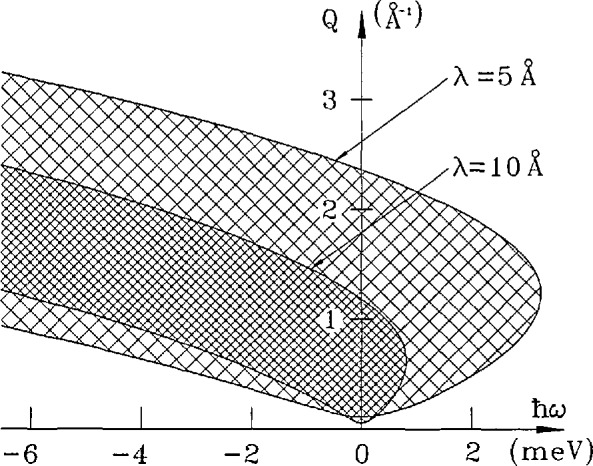
Plots of the accessible region in (*Q*,*ω*) space for neutrons of wavelength 5 and 10 Å (energy 3.272 and 0.818 meV, respectively). The minimum and maximum scattering angles are 5° and 140°. There is no (theoretical) limit to the energy transfer in neutron energy gain.

In Fig. 17 we show accessible regions for two choices of the incident energy. Detectors are assumed to fill the angular range from 5° to 140°.

The accessible region gets smaller as *E*_0_ is decreased. In particular the accessible range of *Q* for elastic scattering (*hω* = 0) is reduced since in this case Eq. (6) simplifies to the well-known (Bragg) relationship

$$Q=2k_{0}\;\sin\left(\phi /2\right).$$ (7)

A decrease in *E*_0_ also means a proportionate reduction in the maximum possible energy transfer in neutron energy loss. The improved resolution that results as *E*_0_ is decreased, i.e., as λ_0_ is increased, is a direct consequence of the contraction in accessible (*Q*,*ω*) space.

### 5.2 Resolution and Intensity

An important contribution to the overall energy resolution of a TOF spectrometer arises from the spread in the time distribution of neutrons in the incident beam.

In the FCS there are two independent sources of incident beam time spread: the monochromator and the chopper. The chopper contribution is independent of distance but the wavelength spread from the monochromator translates into a time spread which increases with distance. The net result is that the width of the time distribution in the unscattered beam, at a point distant *x* from the chopper, may be written as follows:

$$\sigma^{2}\left(x\right)=\sigma_{t}^{2}+x^{2}\sigma_{\tau }^{2},$$ (8)

where *σ_t_*, measures the burst time of the chopper and *σ_τ_* is the spread in the reciprocal velocity distribution of neutrons in the beam.

The time distribution for a two-chopper spectrometer may be similarly written [30]. In this case the time spread is an appropriate average of the burst times *σ*_1_ and *σ*_2_ for the two choppers:

$$\sigma_{t}^{2}=\sigma_{1}^{2}\sigma_{2}^{2}/\left(\sigma_{1}^{2}+\sigma_{2}^{2}\right).$$ (9)

The reciprocal velocity spread σ*_τ_* is given by

$$\sigma_{\tau }^{2}=\left(\sigma_{1}^{2}+\sigma_{2}^{2}\right)/L_{12}^{2},$$ (10)

where *L*_12_ is the distance between the two choppers, and the distance *x* is measured from an “effective chopping point” which is located between the choppers at a distance

$$L_{10}=L_{12}\;\sigma_{1}^{2}/\left(\sigma_{1}^{2}+\sigma_{2}^{2}\right)$$ (11)

from the first chopper. These expressions may be used to calculate the time distribution in the incident beam of the DCS.

Important additional contributions to the overall energy resolution of a TOF spectrometer stem from flight path uncertainties due to the size of the sample and the geometry of the detectors [5].

The count-rate in a TOF experiment is determined by the intensity of neutrons at the sample position, the scattering properties of the sample, and the detecting efficiency of the array of detectors. To some extent the individual experimenter can select the scattering properties of the sample such as its size and shape, and how it is contained. The efficiency of the detector array can generally be increased, without degrading resolution, simply by purchasing more detectors; thus it largely depends on the available budget. The principal challenge in designing a TOF spectrometer is therefore to optimize the “front end” of the instrument, and indeed considerable efforts have been devoted to this task for both of the CNRF spectrometers.

## 6. Concluding Remarks

The time-of-flight spectrometers at the CNRF are the only instruments of their type on the North American continent. Once operational, we expect that they will fill a significant gap in the arsenal of neutron inelastic scattering instruments available to the scientific community. We look forward to a variety of collaborations and interactions with scientists wishing to use these instruments.

## Figures and Tables

**Fig. 18:**
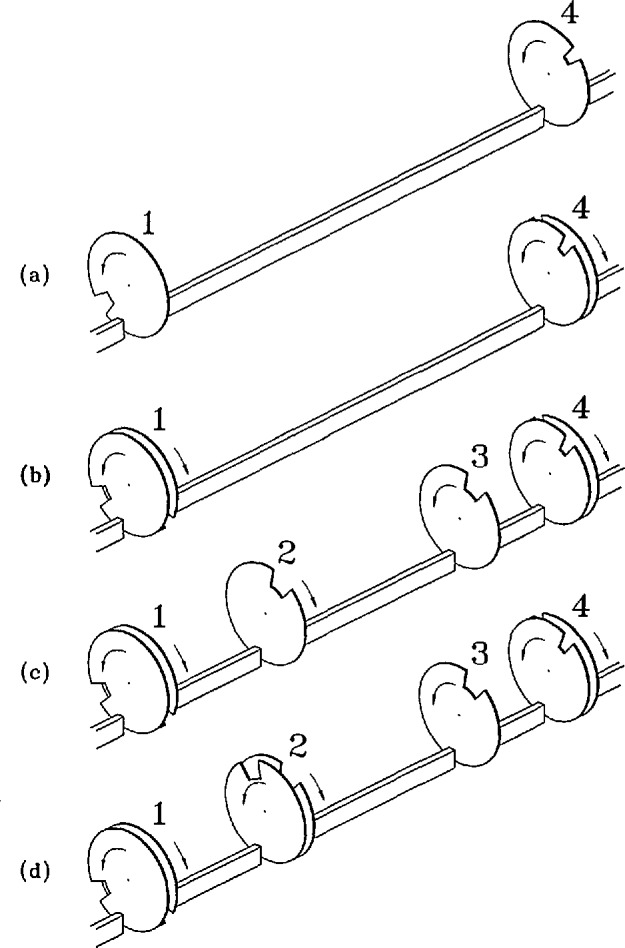
Various chopper arrangements of increasing complexity. At (a) is shown the bare minimum two chopper arrangement. At (b) is shown a system with two counter-rotating choppers. Order removal choppers have been added at (c), and a slow-moving frame removal chopper is included at (d). The symbols 1, 2, 3, and 4 label choppers and chopper pairs.

**Fig. 19:**
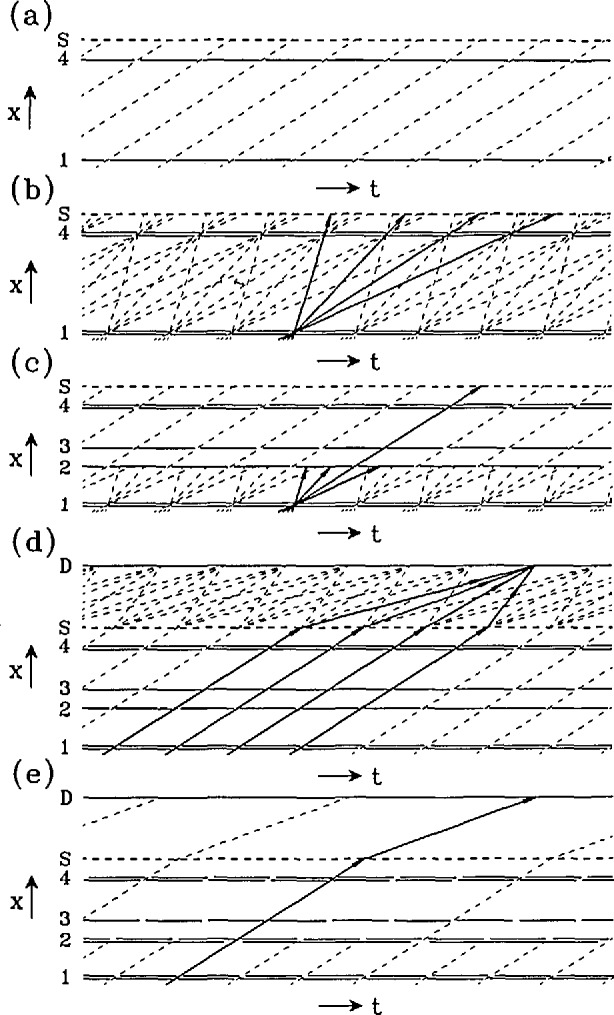
A series of simplified (*x*,*t*) timing diagrams. In each case time is plotted horizontally and distance along the beam direction is plotted vertically; the slope of an inclined line is proportional to the velocity of the corresponding neutrons. The symbols 1, 2, 3, and 4 represent the positions of choppers or chopper pairs; S and D represent the sample position and the detector position, respectively. Breaks in horizontal lines represent time periods when choppers are open to the passage of neutrons. At (a) is shown an idealized timing diagram for a system of two choppers. A more realistic timing diagram for the same system (with counter-rotating choppers) is shown at (b). Several different neutron wavelengths are transmitted and some of those associated with one of the bursts are shown as heavy lines. To stop the unwanted wavelengths order removal choppers are added, as shown at (c). Ambiguities in the analysis of time-of-flight data can arise if the number of bursts at the sample position is too high. This is illustrated at (d); one set of neutrons which arrives at the detector at the same time is shown as heavy lines. A frame removal chopper is used to resolve the problem, as shown at (e); in this example every third burst is transmitted.

**Fig. 20:**
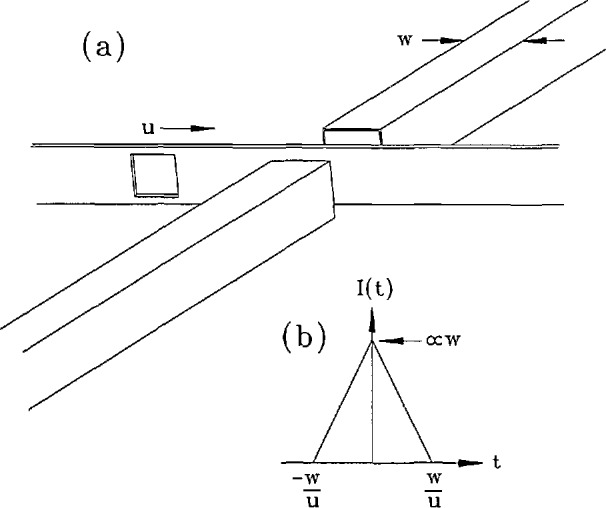
A linear chopping device is illustrated at (a); the widths of the slot and the guide are both *w* and the chopping speed is *u*. The time dependence of the transmitted intensity is shown at (b). The burst time is (*w*/*u*) whereas the transmitted intensity is proportional to (*w*^2^/*u*).

**Table 1:** Specifications and anticipated performance of the Fermi Chopper Spectrometer. Additional details are given in the text

Specifications		
Guide cross section (height × width):	150 × 60 mm	
Maximum beam size (height × width):	100 × 25 mm	
Monochromators (double):	Pyrolytic graphite PG(002)	
1st Monochromator (height × width/type):	160 × 100 mm/flat	
2nd Monochromator (height × width/type):	184 × 100 mm/vertically focussing	
Monochromator mosaic (vertical/horizontal):	25750′	
Intermonochromator collimation:	60′	
Beam filters (thickness/type/temperature):	152 mm/Be/77 K for λ > 0.4 nm	
	38 mm/PG/296 K for select λ <0.4 nm	
Fermi chopper details (2 slit packages)	Package 1	Package 2
Blade curvature radius:	389.0 mm	581.4 mm
Rotor radius:	26.9 mm	25.6 mm
Slot width:	0.635 mm	0.635 mm
Beam window (height × width):	100 × 25 mm	100 × 25 mm
Optimum chopper speed:	80.93/λ(nm)Hz	54.15/λ(nm) Hz
Sample — detector distance:	2.286 m	
Scattering angle range:	5° < *ϕ* < 140°	
Post-Sample radial collimation (oscillating)		
Blade separation:	3°	
Inner/Outer radius:	203/305 mm	
Detectors (cylindrical, ^3^He-filled)		
Fill pressure:	0.4 MPa	
Diameter:	25 mm	
Angular separation:	1.273°	
Quantity/Active height:	12/229 mm for 5° <2*θ* <20°	
	88/457 mm for 20° < 2*θ* < 140°	
Incident wavelength range:	0.23 < λ_0_ 0.61 nm (÷2 for PG(004))	
Incident energy range:	15.5 >*E*_0_ > 2.2 meV (×4 for PG(004))	
Elastic momentum transfer range:	1*< Q* < 50 nm^−1^ (×2 for PG(004))	
Anticipated performance		
Fluence rate on sample:	∼1 × 10^8^ n/m^2^/s at λ =0.24 nm	
	∼1.2 × 10^8^ n/m^2^/s at λ = 0.40 nm	
Elastic energy resolution:	40 *μ*eV at λ = 0.60 nm	
	150 *μ*eV at λ = 0.40 nm	
	600*μ*eV at λ=0.24 nm	
*Q* Resolution:	Δ*Q/Q* < 3%	

**Table 2:** Specifications and anticipated performance of the disk chopper spectrometer. Sets of three quantities within braces refer to the three resolution modes of operation of the instrument, {low, medium, high}, respectively

Specifications				
Beam height at guide exit:				100 mm
Beam width at guide exit:				{30 mm, 15 mm, 5 mm}
Disk chopper details:				
Outside radius:				290 mm
Maximum operating speed:				333 Hz (20,000 rpm)
Maximum tip velocity:				607 m/s
Pulsing chopper slot widths:				{12°, 6.5°, 2.6°}
Monochromating chopper slot widths:				{8°, 3.5°, 1.35°}
Order removal chopper slot widths:				20°, 18°
Frame removal chopper slot width:				20°
Sample-Detector distance:				4 m
Anticipated maximum scattering angle:				140°
Detectors (rectangular cross-section, ^3^He-filled)				
Fill pressure:				0.6 MPa
Width:				−32 mm
Thickness:				~10 mm
Active length:				400 mm
Arrangement:				3 banks
Approximate incident wavelength range:				0.2 < λ_0_ < 1.5 nm
Approximate incident energy range:				20 > *E*_0_ > 0.36 mcV
Corresponding elastic *Q* range:				0.5 < *Q* < 60 nm^−1^
Anticipated performance (assuming a chopper speed of 20,000 rpm)				
Fluence rate on sample			(λ_0_ = 0.2 nm):	{45, 10, 2} × 10^6^ n/m^2^/s
“	“	“	(λ_0_ = 0.4 nm):	{450, 100, 20} × 10^6^ n/m^2^/s
“	“	“	(λ_0_=0.6 nm):	{220, 50, 10} × 10^6^ n/m^2^/s
“	“	“	(λ_0_ = 0.8 nm):	{100, 20, 5} × 10^6^ n/m^2^/s
Elastic energy resolution			(λ_0_ = 0.2 nm):	{1300, 650, 270} u,eV
“	“	“	(λ_0_=0.4 nm):	{160, 80, 35} µeV
“	“	“	(λ_0_ = 0.6 nm):	{50, 25, 12} µeV
“	“	“	(λ_0_ = 0.8 nm):	{21, 11, 6} µeV

## 7. Appendix A

A thermal neutron has energy *E*, wave-vector *k*, wavelength A, velocity *v*, and reciprocal velocity *τ*. These quantities are related as follows:

$$E\left(\text{meV}\right)=\left(h^{2}/8\pi^{2}m\right)k^{2}≃0.020721{\left[k\left({\text{nm}}^{-1}\right)\right]}^{2}$$ (A1)

$$=h^{2}/2m\lambda^{2}≃0.81804/{\left[\lambda \left(\text{nm}\right)\right]}^{2}$$ (A2)

$$=m\upsilon^{2}/2≃5.2270{\left[\upsilon \left({\text{mm}\;\mu s}^{-1}\right)\right]}^{2}$$ (A3)

$$=m/2\tau^{2}≃5.2270/{\left[\tau \left({\mu \text{s mm}}^{-1}\right)\right]}^{2}.$$ (A4)

Furthermore

$$\lambda \left(\text{nm}\right)=h/mv≃0.39560/\left[\upsilon \left({\text{mm}\;\mu s}^{-1}\right)\right],$$ (A5)

and

$$k\left({\text{nm}}^{-1}\right)=2\pi m\upsilon /h≃15.8825\left[\upsilon \left({\text{mm}\;\mu s}^{-1}\right)\right].$$ (A6)

In these equations *m* is the mass of the neutron and *h* is Planck’s constant; values of *m* and *h* are taken from Ref. [31].

Useful conversion factors relating different units of energy may also be found in Ref. [31]. Two of the most commonly employed conversions are the following:

$$\begin{array}{c} 1\;\text{meV}≃8.0655{\text{cm}}^{-1}\\ \;≃0.2418\times 10^{12}\;\text{Hz}. \end{array}$$

## 8. Appendix B

In the disk chopper spectrometer the functions of puiser and monochromator are assumed by a set of disk choppers which rotate about a common axis parallel to (and some distance above) the direction of the beam. The essential concept can be understood by considering the two chopper arrangement illustrated in Fig. 18(a). The pulsing chopper produces short bursts of neutrons of many different energies. These neutrons have different speeds and therefore arrive at the monochromating chopper at many different times. The phasing of the monochromating chopper is chosen to transmit neutrons of the desired energy. The operation of this system can be represented in a distance-time (*x*,*t*) diagram as shown in Fig. 19(a).

Each of the principal choppers in the DCS is actually a counter-rotating pair of disks, as shown in Fig. 18(b). The effective chopping speed for such a device is double that of a single disk which rotates at the same speed as one of the members of the counter-rotating pair [32]. It follows that the intensity per pulse is doubled at constant resolution. This can be understood by considering systems such as the one shown in Fig. 20(a). The burst time of the illustrated system is simply *Δt* = (*w/u*) whereas the transmitted intensity per pulse, *I*_p_, is proportional to (*w*^2^/*u*), where *w* is the width of the slot and the guide, and *u* is the chopping speed; the time dependence of the intensity is illustrated in Fig. 20(b). Clearly

$$I_{P}\propto u{\left(\Delta t\right)}^{2}$$ (B1)

so the intensity per pulse, at constant burst time, is proportional to the chopping speed of the system. A further advantage of the counter-rotating chopper pair concept, the possibility of using slots of different widths to enhance the resolution capabilities of the instrument [29], is discussed in Sec. 4.2. The timing diagram for two counter-rotating chopper pairs is essentially of the type illustrated in Fig. 19(a).

Up to now we have glossed over the fact that neutrons of more than one wavelength can be transmitted by systems such as those shown in Figs. 18(a) and 18(b); this possibility is illustrated in Fig. 19(b). Neutrons of the desired wavelength (λ_0_) take a certain time (which is proportional to λ_0_) to travel the distance between the choppers. Neutrons which take an integral number of chopper periods longer (or shorter) than this time to travel the same distance stand an equal chance of being transmitted. To suppress such neutrons we have determined that two additional “order removal” choppers are required. These choppers are placed at intermediate locations between the pulsing and monochromating choppers as shown in Figs. 18(c) and 19(c); their exact positions are critical in order to remove all orders with intensity above background, under all conceivable operating conditions of the instrument.

The six choppers so far described collectively produce sharp bursts of neutrons of a single well defined energy at the sample position, as in Fig. 19(c). From our previous discussion we have seen that these choppers should be operated at the highest acceptable speed in order to achieve the highest possible intensity per pulse, but unfortunately the shortness of the time between pulses can introduce ambiguities into the analysis of a TOF measurement. This is illustrated in Fig. 19(d). We see that neutrons which arrive at the detector at a given time can in general be associated with more than one pulse arrival time at the sample. On physical grounds it is generally possible to rule out all but a few possible energy transfers, but ambiguities may still remain, in which case the appropriate course of action is to reduce the number of pulses at the sample. This is achieved using a chopper which rotates with a period that is an exact multiple of the period of the other choppers. The net effect is illustrated in Fig. 19(e). In the DCS the frame overlap chopper is placed close to the first order removal chopper, as shown in Fig. 18(d), and in Fig. 15.
